# Cytokine Signature Induced by SARS-CoV-2 Spike Protein in a Mouse Model

**DOI:** 10.3389/fimmu.2020.621441

**Published:** 2021-01-28

**Authors:** Tingxuan Gu, Simin Zhao, Guoguo Jin, Mengqiu Song, Yafei Zhi, Ran Zhao, Fayang Ma, Yaqiu Zheng, Keke Wang, Hui Liu, Mingxia Xin, Wei Han, Xiang Li, Christopher D. Dong, Kangdong Liu, Zigang Dong

**Affiliations:** ^1^Department of Pathophysiology, School of Basic Medical Sciences, Academy of Medical Science, College of Medicine, Zhengzhou University, Zhengzhou, China; ^2^China-US (Henan) Hormel Cancer Institute, Zhengzhou, China; ^3^Affiliated Cancer Hospital of Zhengzhou University, Zhengzhou, China; ^4^The Henan Luoyang Orthopedic Hospital, Zhengzhou, China; ^5^Henan Provincial Cooperative Innovation Center for Cancer Chemoprevention, Zhengzhou University, Zhengzhou, China; ^6^Wartburg College, Waverly, IA, United States

**Keywords:** COVID-19, SARS-CoV-2, acute respiratory distress syndrome, murine model, cytokine storm syndrome

## Abstract

Although COVID-19 has become a major challenge to global health, there are currently no efficacious agents for effective treatment. Cytokine storm syndrome (CSS) can lead to acute respiratory distress syndrome (ARDS), which contributes to most COVID-19 mortalities. Research points to interleukin 6 (IL-6) as a crucial signature of the cytokine storm, and the clinical use of the IL-6 inhibitor tocilizumab shows potential for treatment of COVID-19 patient. In this study, we challenged wild-type and adenovirus-5/human angiotensin-converting enzyme 2-expressing BALB/c mice with a combination of polyinosinic-polycytidylic acid and recombinant SARS-CoV-2 spike-extracellular domain protein. High levels of TNF-α and nearly 100 times increased IL-6 were detected at 6 h, but disappeared by 24 h in bronchoalveolar lavage fluid (BALF) following immunostimulant challenge. Lung injury observed by histopathologic changes and magnetic resonance imaging at 24 h indicated that increased TNF-α and IL-6 may initiate CSS in the lung, resulting in the continual production of inflammatory cytokines. We hypothesize that TNF-α and IL-6 may contribute to the occurrence of CSS in COVID-19. We also investigated multiple monoclonal antibodies (mAbs) and inhibitors for neutralizing the pro-inflammatory phenotype of COVID-19: mAbs against IL-1α, IL-6, TNF-α, and granulocyte-macrophage colony-stimulating factor (GM-CSF), and inhibitors of p38 and JAK partially relieved CSS; mAbs against IL-6, TNF-α, and GM-CSF, and inhibitors of p38, extracellular signal-regulated kinase, and myeloperoxidase somewhat reduced neutrophilic alveolitis in the lung. This novel murine model opens a biologically safe, time-saving avenue for clarifying the mechanism of CSS/ARDS in COVID-19 and developing new therapeutic drugs.

## Introduction

In early December 2019, cases of viral pneumonia of unknown cause appeared in Wuhan, China. The causative viral strain was first isolated from patient samples on January 7, and the entire viral genome sequence was obtained by January 10, 2020 (1, 2). The virus showed homology of 79% and 50% to the genomes of SARS-CoV and MERS-CoV, respectively, and was thus named as the novel coronavirus SARS-CoV-2 (3). The illness known as COVID-19 was demonstrated to be caused by SARS-CoV-2 infection (4), and a total of 78,679,912 confirmed cases of COVID-19 had been reported globally as of December 22, 2020. The total number of deaths worldwide has exceeded 1 million, even the BioNTec/Pfizer vaccine has now been approved in Europe, but for exists COVID-19 patients, and we also need more efficacious antiviral drugs. The COVID-19 pandemic is a threat to public health, and methods to control the spread of the virus and improve treatment have become urgent issues concerning the national security of governments all around the world.

As the course of COVID-19 progresses, patients may develop acute respiratory distress syndrome (ARDS) and cytokine storm syndrome (CSS) (5). CSS, which reflects a disordered immune system and involves a rapid increase of pro-inflammatory cytokine levels in response to a stimulus, is associated with the deterioration of various severe diseases (6). In COVID-19 patients, CSS is marked by significant increases in the plasma levels of pro-inflammatory cytokines including IL-6, IL-1β, IL-2, IL-8, IL-17, granulocyte colony-stimulating factor (G-CSF), granulocyte-macrophage colony-stimulating factor (GM-CSF), IP-10, monocyte chemoattractant protein (MCP)-1, macrophage inflammatory protein (MIP)-1α, and tumor necrosis factor (TNF)α, which leads to the recruitment of immune cells through a positive feedback loop that eventually forms a cytokine storm (7–9). Clarifying the mechanism of CSS in COVID-19 and identifying the key cytokines involved would contribute to the development of more effective methods to block CSS and would be of great significance for the treatment of severe cases. The continuation of the COVID-19 pandemic across the globe emphasizes the need to establish an animal model of COVID-19-related pneumonia. So far, a human angiotensin-converting enzyme 2 (ACE2) transgenic mouse model and an adenovirus-assisted human ACE2 transduction mouse model have been used to study the disease process of COVID-19. Although these models partially mimic the pathologic process of COVID-19, their utility has been hampered by low virus replication efficiency and a lack of obvious symptoms. In the COIVID-19 murine model, the lacks of biosecurity become the most challenging to scientists, SARS-CoV-2 in animal research requires an appropriate biosafety level (BSL3) to avoid the potential leakage risks from laboratory to environment (10, 11). Therefore, there is an urgent need to establish an effective animal research model that mimics the pathological processes of ARDS and CSS in COVID-19. More importantly, the mouse model should be biologically safe, with no risk of infection to research personnel.

We describe here the successful development of a highly biologically safe ARDS animal model that simulates the CSS and the pathophysiological changes that occur in COVID-19 patients. Using polyinosinic-polycytidylic acid (poly[I:C]) combined with a recombinant SARS-CoV-2 spike-extracellular domain protein (SP) as an immunostimulant to mimic SARS-CoV-2, we found that the cytokine signature in bronchoalveolar lavage fluid (BALF) showed high similarity with clinical outcomes. We also identified neutralizing monoclonal antibodies (mAbs) and inhibitors that have prophylactic efficacy for COVID-19. Most importantly, this mouse model may be useful for the development of intervention strategies for both the current pandemic and future infectious diseases caused by coronaviruses.

## Methods and Material

### Experimental Animals

Male BALB/c mice (8–10 weeks) free of pathogens were purchased from Beijing Vital River Laboratory Animal Technology Co., Ltd. (Beijing, China). Mice were housed in a pathogen-free environment under conditions of 20°C ± 2°C, 50% ± 10% relative humidity, 12 h light/dark cycles. They were provided with food and water *ad libitum*. All experimental procedures involving animals were approved by the Ethics Review Commission of Zhengzhou University (following internationally established guidelines, ID-CUHCI2020001).

### Adenovirus Delivery

Adenovirus encode human ACE2 was purchased from Hanheng Biotechnology (Hanheng Biotechnology Co., Ltd., Shanghai, China). Mock adenovirus encodes EGFP was used as a control adenovirus; all of recombinant adenovirus were based on type 5 (E1/E3 deficient) adenovirus. Viral titer was 3.16*10^11^ PFU/ml for *in vivo* infection of human ACE2, deliver of adenovirus through nasal, intravenous or intratracheal injection.

### Surface Plasmon Resonance Assay

The affinity of recombinant SARS-CoV-2 spike-ECD protein with human or mouse ACE2 recombinant proteins was measured by surface plasmon resonance experiments, BIACORE T-200 (GE Healthcare, UK), which were performed with a research-grade CM5 sensor chip (GE Healthcare, UK). Recombinant SARS-CoV-2 spike-ECD protein (Cat# Z03481, GenScript, Nanjing, China) was immobilized using an amine-coupling kit at a value of 200 RU (response unit). To calculate the affinity, the human ACE2 recombinant protein (Cat#10084-H08H, Sino Biological, Beijing, China) or mouse ACE2 recombinant protein (Cat# 50249-M08H, Sino Biological, Beijing, China) in PBS (pH 7.4) was injected at concentrations ranging from 0.18 μM to 2.9 μM. For each sample sensorgram, the relative response was collected and the blank subtracted.

### *Ex Vivo* Binding Assay

The plasmids encoding human ACE2 and mouse ACE2 were purchased from YouBio Company (YouBio, Changsha, China). Transient transfection of HEK293T cells were performed using Xfect transfection reagent (Clontech). After 24-h transfection, HEK293T cells were harvested for *ex vivo* binding assay, recombinant SARS-CoV-2 spike-ECD proteins (0.8 mg/ml) were added into HEK293T cells at 100-μl PBS, volume of SP ranging from 1 to 10 μl, incubate for 1 h. Anti-DYKDDDK-APC antibody (BioLegend, #637308) was used for detect the SP in HEK293T cell surface, Rat-IgG2a-APC as control.

### Western Blot Analysis

Anti-ACE2 (1:1,000, Cat#21115-1-AP, Proteintech, Wuhan, China), anti-FLAG (1:1,000, Cat#F1808, Sigma-Aldrich, Shanghai, China), anti-GAPDH (1:2,000, Cat#HRP-60004, Proteintech, Wuhan, China), and anti–β-actin (1:2,000, Cat#HRP-60008, Proteintech, Wuhan, China) antibodies were performed for western blotting, then with secondary antibodies labeled with HRP and detected by ECL.

### COVID-19 ARDS Murine Model and Treatment

Mice were anesthetized *via* intraperitoneal (IP) injection with Pentobarbital Sodium (50 mg/kg). A small incision was made over the trachea, and the underlying muscle and glands were separated to expose the trachea. Mice were intratracheally administered with freshly mixed poly(I:C) poly[I:C]-HMW, Invivogen, tlrl-pic) 2.5 mg/ml and SARS-CoV-2 recombinant spike protein (SP) (ECD-His-tag, Genescript, Z03481) 15 μg (in saline), followed by 100-µl air, 2.5 mg/kg poly (I:C), FC control (ACRO, P01857-1), 15 μg SARS-CoV-2 recombinant SP, and saline were administered intratracheally independent at the same volume as control. The level of endotoxin contamination in Saline, poly(I:C), FC, and rSP was determined with a Chromogenic LAL Endotoxin Assay Kit (Cat# L00350C, Genscript).

Blocking and neutralizing antibodies anti–IL-1α (InVivoMab, BE0243), IL-6R (InVivoMab, BE0047), TNF-α (InVivoMab, BE0058), IL-6 (InVivoMab, BE0046), GM-CSF (InVivoMab, BE0259), and TNFR2 (InVivoMab, BE0247) were administrated intraperitoneally as a single dose of 200 µg 24 h in prior. TLR3/dsRNA inhibitor (Merck, 614310) was administrated *via* i.p. at 50 mg/kg 2 h prior to administration of SARS-CoV-2 mimics. MPO inhibitor (Merck, A41909) was administrated *via* i.p. at 50 mg/kg per day, 3 days prior to administration of SARS-CoV-2 mimics. P38 inhibitor (MCE, HY-10256) was administrated *via* i.p. at 20 mg/kg per day, 3 days prior to administration of SARS-CoV-2 mimics. ERK inhibitor (MCE, HY-19696A) was administrated *via* i.p. at 100 mg/kg per day, 3 days prior to installation of SARS-CoV-2 mimics. Elastase Inhibitor (GLPBIO, GC11981) was administrated *via* i.p. at 5 mg/kg per day, 3 days prior to administration of SARS-CoV-2 mimics. JAK inhibitor (MCE, HY-15315) was administrated *via* i.p. at 20 mg/kg per day, 3 days prior to administration of SARS-CoV-2 mimics.

### Analysis of BAL Samples

Six hours after the administration, mice were anesthetized *via* IP injection of Pentobarbital Sodium 50 mg/kg. A 26G venous indwelling needle hose was inserted into the exposed tracheal lumen, and then, the airway was washed three times with 1-ml saline each, the first lavage fluid sample was kept separately to test cytokines.

### ELISA Assay

Levels of soluble mouse E-selectin, VCAM-1, and VEGF in BALF or serum were determined using ELISA kits for Mouse Selectin, Endothelium (SELE) ELISA Kit (DongLin Sci&Tech, China), Mouse Vascular Cell Adhesion Molecule 1 (VCAM1) ELISA Kit (DongLin Sci&Tech, China), and serum were verified by Mouse VEGF ELISA Kit (MultiSciences, Hangzhou, China). The ELISA assay was performed to detect the concentration of those protein following the manufacturer’s instructions.

### Multiplex Cytokines Assay

BAL was collected from mice lung after anesthetized, the mice BAL were separated by centrifuging and stored at −80°C. BAL sample collection was performed as previously described. Next, concentrations of mice inflammatory related cytokines IL-1α, IL-1β, IL-6, IL-10, IL-12p70, IL-17A, IL-23, IL-27, MCP-1, IFN-β, IFN-γ, TNF-α, and GM-CSF were measured by LEGENDplex™ Mouse Inflammation Panel (Biolegend, 740446), the data were harvest by flow cytometry using FACSCalibur (BD).

### Flow Cytometry Analysis

Fluorescent-labeled antibodies were performed to quantify neutrophils and macrophages. Unconjugated anti-mouse CD16/CD32 (Biolegend, 101320) was used for blocking Fc receptors, APC-labeled anti-mouse Ly-6G/Ly-6C (Gr-1) (Biolegend, 108412) and FITC-labeled anti-mouse F4/80 (BioLegend, 123108) were performed for neutrophils and macrophages, incubated 30 min on ice, protect from light, washed three times by PBS, and centrifuged to remove the supernatant and responded in 150-µl PBS. Samples were analyzed in the BD FACSCalibur (BD).

### Histopathology Analysis

An independent experiment was performed for the histopathology analysis of lung. The mouse lungs were removed intact and weighted, then fixed in 10% formalin, and paraffin-embedded. Three different fields from a lung section were evaluated; 3-µm sections were sliced on a Leica model rotary microtome and stained with hematoxylin-eosin. Histological analysis was subjected by two independent skilled pathologists, in double-blind.

### Mouse Macrophage Cell Culture

RAW264.7 cell was cultured in DMEM medium supplemented with penicillin (100 units/ml), streptomycin (100 μg/ml), and 10% FBS (Biological Industries, Kibbutz Beit-Haemek, Israel).

### Compounds Treated to RAW264.7

RAW264.7 cells (6 × 10^5^ cells/well) were seeded into 12-well plates and cultured for 24 h in incubator. The cells were treated with a series dilution of compound X; poly(I:C) (10 μg/ml) were added into medium in a 12-well plate. Meanwhile, RAW264.7 cell was subjected to DMEM with or without poly(I:C) as a positive or negative control. The cells were maintained at 37°C in a 5% CO_2_ incubator for 12 h, harvested the supernatant for the detection of IL-6 secretion by Mouse IL-6 Elisa KIT(MULTISCIENCES, 70-EK201BHS-96).

### Mouse Primary Macrophage Cell Culture

Bones were gently separated from mouse, and the connecting muscles and soft-tissues were all removed from the bone. The macrophages contain in the bone were washed out by DPBS using 1-ml syringe. Suspended the eluate and centrifuged at 1,000 rpm for 5 min. Washed the pellets by DPBS and resuspended the cell pellets by RPMI-1640 containing penicillin (100 units/ml), streptomycin (100 μg/ml), 10% FBS (Biological Industries, Kibbutz Beit-Haemek, Israel), and 50 ng/ml M-CSF (peprotech), plated the cells in a low attachment culture dish and maintained at a 5% CO_2_ incubator. Renew the medium of fresh RPMI-1640 complete medium contained 50 ng/ml M-CSF every other day until the density of adherent cells reached 90%. Digested the cells and plated 6×10^5^ cells/well into 12-well plates, cultured for 24 h and treated the poly(I:C) and compound to the cell using blank RPMI-1640 medium. This method is similar to the treatment of RAW264.7.

### Dissociated of Primary Lung Cells Mix

Took out the whole lung tissues of the mice and washed by pre-cold PBS containing DNase I (0.01 mg/ml) (Sigma), penicillin (100 units/ml), and streptomycin (100 μg/ml). Tattered the lung tissues into small pieces in a sterile centrifuge tube by scissors, suspended the small pieces by 1 mg/ml Type I collagenase (Sigma) supplied by 0.01 mg/ml DNase I and digested in a 37°C shaking incubator for 30 min. Filtered the solution with a 200 mesh sieve and centrifuged at 1,000 rpm for 5 min, subsequently. After washed the pellets twice by pre-cold PBS containing DNase I (0.01 mg/ml), resuspended the pellets by RPMI-1640 containing 10% FBS (Biological Industries, Kibbutz Beit-Haemek, Israel) and seeded into 12-well plate. Harvested the supernatant after 24 h culture and removed the floating cells by centrifuge at 3,000 rpm for 5 min. The ELISA assay (Mouse IL-6 Elisa KIT, MULTISCIENCES, 70-EK201BHS-96) was performed to detect the IL-6 concentration following the manufacturer’s instructions.

### Statistical Analysis

Statistical analysis was performed using GraphPad Prism 7.0 (GraphPad Software, United States). Specific statistical methods and comparisons made by methods as described in figure legend. Comparison between two groups were performed by unpaired Student’s t-test. Multiple comparisons were performed using one-way ANOVA with Bonferroni or Dunnet *post hoc* test, as appropriate. *P* < 0.05 was regarded as statistically significant and marked with a star, data was reported as mean ± SEM, and error bars indicate SEM.

### Graphical Illustrations

Schematic illustrations were established with BioRender (BioRender.com).

## Results

### Poly(I:C) + SP-Induced ARDS in BALB/c Mice

**Figure 1:**
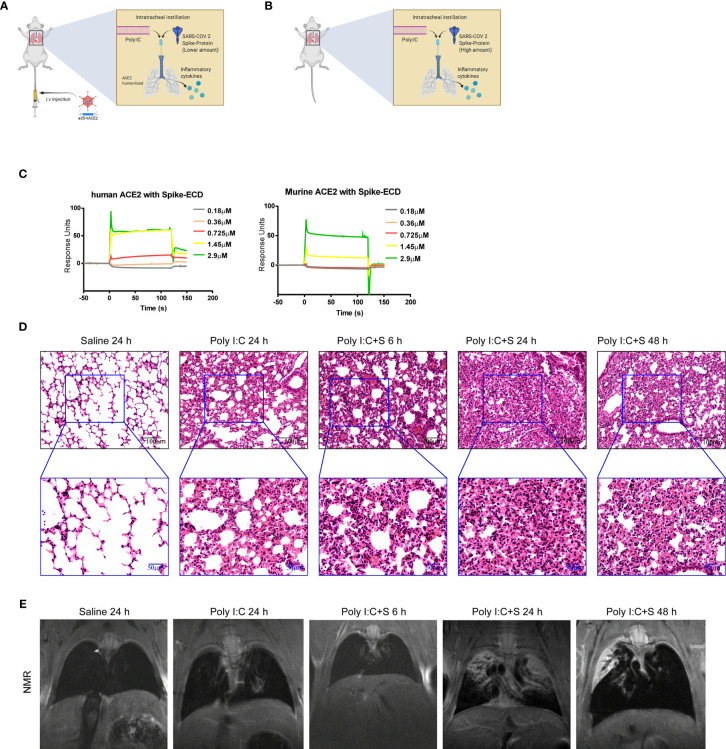
SARS-CoV-2 mimic–induced acute lung injury in BALB/c mice. Schematic diagrams of the delivery of polyinosinic-polycytidylic acid (poly[I:C]) + recombinant SARS-CoV-2 spike-extracellular domain protein (ECD) (poly[I:C] + SP) by intratracheal administration to **(A)** adenovirus 5-human angiotensin-converting enzyme 2 (ad5-hACE2) transgenic and **(B)** wild-type Balb/c mice. **(C)** Surface plasmon resonance sensorgrams of transgenic (left panel) and wild-type (right panel) mice. **(D)** Histologic characteristics of lung injury and interstitial pneumonia induced by poly(I:C) + SP at 6, 24, and 48 h after challenge. Black scale bar = 100 µm; blue scale bar = 50 µm. **(E)** In vivo small animal magnetic resonance images documenting the pleural effusion induced by poly(I:C) +SP at 6, 24, and 48 h after challenge. Control group mice (Saline) received saline + poly(I:C) challenge and were assessed at 24 h.

To explore the pathogenesis of SARS-CoV-2 components in the cytokine-release storm that is observed in COVID-19 (12), we sought to generate a mouse model that would mimic SARS-CoV-2 infection and induce COVID-19 disease. Coronaviruses possess a large RNA genome and can stimulate host toll-like receptor (TLR)3 and TLR7 upon infection (13–17). We chose poly(I:C) to mimic the effect of viral RNA. First, we established a line of BALB/c mice expressing humanized ACE2 through infection with an adenovirus-based vector. The lungs of these mice were then inoculated with the SARS-CoV-2 mimic poly(I:C) + SP *via* intratracheal injection (**Figure 1A**). We also inoculated the lungs of wild type Balb/c mice *via* intratracheal injection of poly(I:C) + SP (**Figure 1B**). To confirm that the recombinant SARS-CoV-2 SP could bind with mouse ACE2, a surface plasmon resonance technology (SPR) assay was performed. SPR sensorgrams showed an increase in SPR signal response units with increasing levels of human and mouse ACE2 recombinant proteins; the affinity of mouse ACE2 for binding to SP was detectable, but was lower than that of human ACE2 (**Figure 1C**). The affinity of mouse ACE2 for SP showed the potential for the mimicking of COVID-19 pathogenesis using high-dose SARS-Cov-2 virus or SP in mice, when compared with ACE2 humanized mouse model. When the administered dose of recombinant SP in our SARS-CoV-2 mimic was increased, we observed acute lung injury featuring neutrophilic inflammation and interstitial edema. Furthermore, the lung tissue showed more severe injury after injection of poly(I:C) + SP compared with poly(I:C) induced alone. To better understand the time course of pathologic changes, we examined the injury to mouse lungs at different timepoints. Lung injury was apparent at 6 h and was most severe at 24 h, but had gradually decreased at 48 h (**Figure 1D**). The mice also suffered from extensive pleural fluid accumulation between 24 and 48 h post-challenge. Pleural effusion was measured by small animal magnetic resonance imaging. Gross lung lesions that were observed at 24 h were reduced at 48 h post-challenge; these lesions were not apparent at 6 h (**Figure 1E**).

### Poly(I:C) + SP-Induced ARDS in ACE2 Humanized Mice

**Figure 2:**
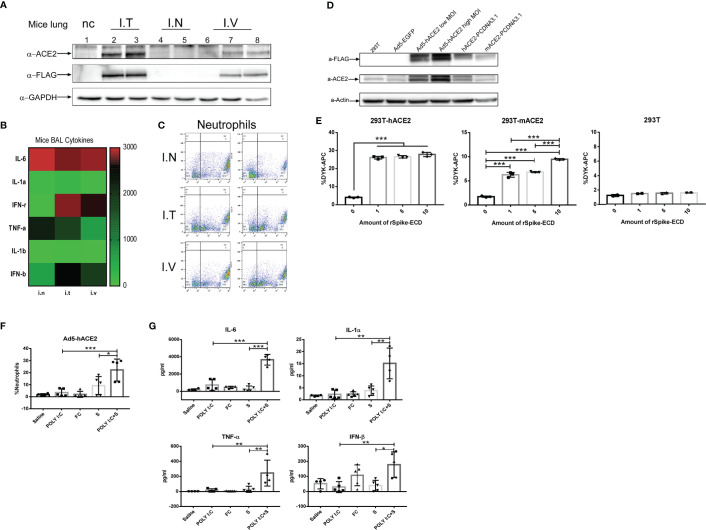
Development of angiotensin-converting enzyme 2-humanized (hACE2) mice sensitized to SARS-CoV-2 mimic challenge. **(A)** Western blot assay of hACE2 expression using antibodies against GAPDH, ACE2 and the DYKDDDDK (FLAG) tag. Five days post virus transduction, mice were challenged with SARS-CoV-2 mimic *via* three different deliver methods: nasal (I.N.), intratracheal (I.T.), and intravenous (I.V) injection. Inflammation-related cytokines in bronchoalveolar lavage fluid (BAL); α-FLAG and α-GAPDH cropped from different parts of the same gel, but different exposures, α-ACE2 cropped from different gels **(B)** and neutrophil infiltration **(C)** in hACE2 mice. **(D)** Western blot assay in HEK293T cells transduced with adenovirus 5 (Ad5)-hACE2 or Ad5-EGFP at a multiplicity of infection (MOI) of 25 or 50, or transiently transfected with PCDNA3.1 carrying hACE2 or mouse ACE2 (mACE2), using antibodies against β-actin (control), ACE2, and the FLAG tag; α-ACE2 and α-actin cropped from different parts of the same gel, but different exposures, α-FLAG cropped from different gels. **(E)** Recombinant SARS-CoV-2 spike-extracellular domain protein (rSpike-ECD) was incubated with HEK293T cells transiently transfected with hACE2 or mACE2 for 1 h at room temperature and detected by flow cytometry using allophycocyanin (APC)-labeled anti-DYKDDDDK antibody. Neutrophil infiltration **(F)** measured by flow cytometry and inflammatory-related cytokine profile **(G)** after challenge with SARS-CoV-2 mimic (poly[I:C] + SP), poly(I:C) alone, S alone, saline control (each n ≥ 4), *via* nasal injection at 5 days post virus transduction). *P < 0.05; **P < 0.01; ***P < 0.001, one-way ANOVA with a *post hoc* Bonferroni test.

To establish ACE2-humanized mice, three different approaches were selected to deliver an adenovirus-based construct carrying human ACE2 with a DYKDDDDK (FLAG) tag. Exogenous expression of human ACE2 and the FLAG tags was detected in mouse lung tissue 5 days following intratracheal and intravenous injection, but not nasal injection, of the construct (**Figure 2A**). Examination of mouse BALF also revealed significant increases in the production of inflammation-related cytokines IL-6 and TNF-α (**Figure 2B**) and neutrophil infiltration (**Figure 2C**). HEK293T cells were infected with the adenovirus 5-human ACE2 (Ad5-hACE2) virus at both a low (25) and high (50) multiplicity of infection. Compared with uninfected HEK293T cells and an Ad5-enhanced green fluorescent protein (EGFP) control group, western blotting of cells infected with Ad5-hACE2 showed positive bands specific for antibodies against ACE2 and FLAG (**Figure 2D**). We also transiently transfected HEK293T cells with human or mouse ACE2 then incubated the cells with 1-, 5-, or 10-µl SP (0.8 mg/ml, FLAG-tagged). Flow cytometry revealed a dose-dependent increase in the detection of FLAG-bearing cells (**Figure 2E**). These results showed that our recombinant SARS-CoV-2 SP could bind with the endogenous mouse ACE2 receptor *in vitro* and *ex vivo*, although the affinity for mouse ACE2 was lower than that for human ACE2. Furthermore, when we compared the immunochallenge of the SARS-CoV-2 mimic with controls in ACE2-humanized mice, the infiltration of neutrophils in SARS-CoV-2 mimic–challenged mice was significantly higher than that in any of the single-challenge groups (poly[I:C], SP, FC; **Figure 2F**). The production of inflammatory cytokines in this model showed a similar tendency; that is, the levels of IL-6, IL-1α, IFN-β, and TNF-α in mimic-challenged mice were significantly higher than those in the single-challenge poly(I:C), FC, and SP groups (**Figure 2G**).

### Cytokine Profile in Poly(I:C) + SP-Induced ARDS

**Figure 3:**
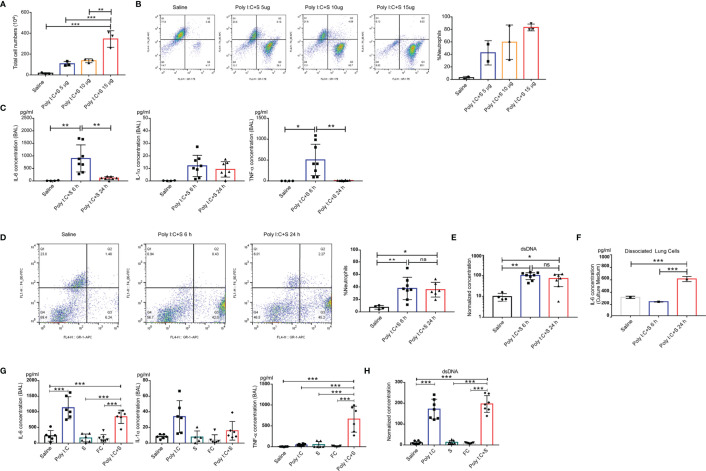
Polyinosinic-polycytidylic acid (poly[I:C]) + recombinant SARS-CoV-2 spike-extracellular domain protein (SP)-induced cytokine-release storm in the mouse lung. **(A)** Total cell count in the BAL from poly(I:C) + 5-, 10-, or 15-µg SARS-CoV-2 spike protein-challenged mice; control group, Saline (each n ≥ 3); **(B)** Cellular composition in BAL. **(C)** Production of IL-6, IL-1α, and tumor necrosis factor (TNF)α in the bronchoalveolar lavage fluid (BAL) from poly(I:C) + SP-challenged mice. 6 or 24 h after SARS-CoV-2 mimic challenged mice. Control group; Saline (each n ≥ 4); **(D)** percentage of neutrophils in BAL; **(E)** Levels of dsDNA in the BAL. Single cells dissociated from the lung tissue of poly(I:C) + SP-challenged mice. **(F)** IL-6 concentration from cell culture medium. **(G)** Concentrations of IL-6, IL-1α, and tumor necrosis factor (TNF)α in bronchoalveolar lavage fluid (BALF) after challenge with SARS-CoV-2 mimic (poly[I:C] + SP), poly(I:C) alone, SP alone, FC alone, saline control (each n ≥ 5), **(H)** Levels of dsDNA in the BALF. *P < 0.05; **P < 0.01; ***P < 0.001, ns (non-significant), p > 0.05, one-way ANOVA with a *post hoc* Bonferroni test.

To understand the molecular pathology of poly(I:C) + SP-induced lung injury, cytokine profiles and flow cytometric analysis were performed to examine the involvement of inflammatory molecules and related immune cells. Total cellularity of mouse BALF samples showed a significant increase, in an SP dose-dependent manner, after 24 h challenge (**Figure 3A**). We further investigated the cellularity in BALF samples using flow cytometry, which showed that neutrophils, but not macrophages, significantly infiltrated and migrated into the lungs. It should be noted that the increase in the number of neutrophils in the BALF was SP dose-dependent compared with saline (**Figure 3B**). For the timepoint study, the SARS-CoV-2 mimic led to an increase in the levels of inflammatory cytokines IL-1α, IL-6, and TNF-α at 6 h; however, TNF-α and IL-6 decreased significantly at the 24-h timepoint compared with the 6 h timepoint. Compared with saline, the levels of all cytokines were increased at the 6 h timepoint (**Figure 3C**). We noted no significant difference in the infiltration of neutrophils between the 6 and 24 h timepoints (**Figure 3D**). Moreover, a unique mechanism of the neutrophil effector is the generation of NETs; the level of NETs can be quantified by measuring cell-free dsDNA. We used the concentration of dsDNA as an indicator of NETs. At the 6 and 24 h post-challenge timepoints, no significant difference was observed in the concentration of dsDNA in mouse BALF samples (**Figure 3E**). We also dissociated cells from mouse lung tissue after 24 h instillation of poly(I:C) + SP, and maintained them in culture medium for 6 and 24 h. The concentrations of IL-6 continued increasing after dissociation, and the cells secreted more IL-6 into the medium with the longer period of maintenance. This indicated that CSS may occur in the lungs following instillation of SARS-CoV-2, along with highly inflammatory and activated immune cells such as tissue-resident macrophages (**Figure 3F**). The levels of IL-6 and TNF-α were dramatically increased in the poly(I:C) + SP group compared with the single poly(I:C) and SP control groups. Saline and recombinant FC protein did not stimulate the production of cytokines, while poly(I:C) or SP showed higher levels of IL-1α, IL-6, and TNF-α (**Figure 3**). Moreover, intratracheal injection of saline, SP or recombinant FC protein did not stimulate an increase in double-stranded DNA (dsDNA), an indicator of neutrophil extracellular traps (NETs; **Figure 3G**). The endotoxin contamination in Saline, poly(I:C), FC, and SP were verified using a LAL (Limulus Amoebocyte Lysate) test (**Supplementary Figure 2**). Markers of the endothelial damage including soluble mouse E-selectin, VCAM-1, and VEGF were tested by ELISA assay in both mouse BALF and serum samples (**Supplementary Figure 1**), the activation of endothelial in mouse lung during the challenge of poly(I:C) and SP was indicated by VEGF production in BALF. VEGF in BALF was increased significantly post administration of poly(I:C) and SP, this result may indicate that the activation of endothelial may induced by SP. The integrity of endothelial in mouse lung was verified by soluble mouse E-selectin, VCAM-1 in mouse serum, we can notice the significantly increased soluble mouse VCAM-1 in poly(I:C) and SP challenged group compared with Saline or FC group.

### Evaluation of the Roles of Neutralizing Antibodies in SARS-CoV-2 Mimic–Induced ARDS

**Figure 4:**
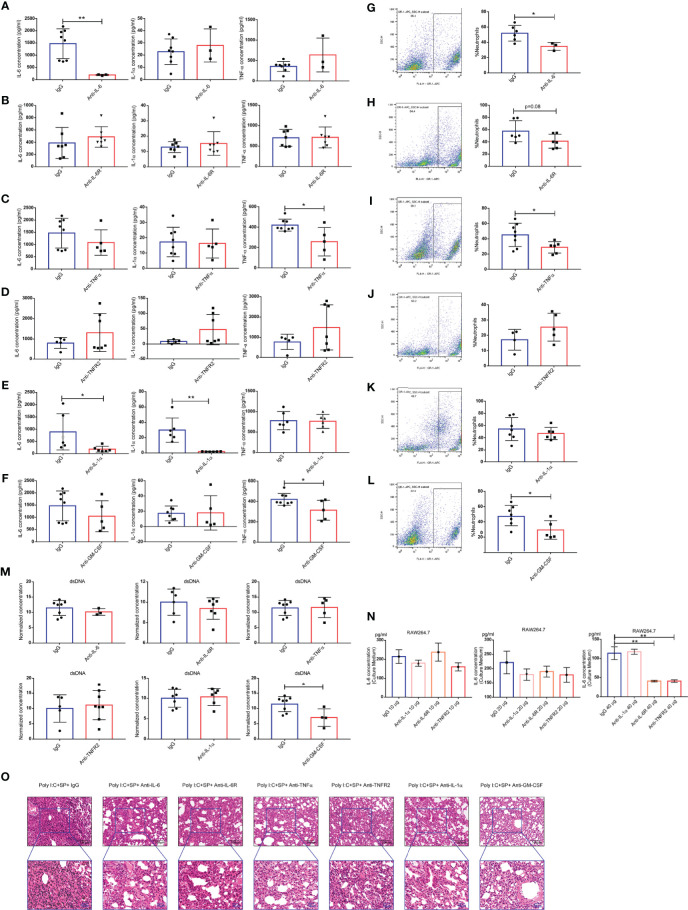
Neutralization and blocking of inflammatory cytokines. Concentrations of IL-6, IL-1α, and tumor necrosis factor (TNF)α in bronchoalveolar lavage fluid (BALF) of mice after treatment with the following monoclonal antibodies (mAbs): anti–IL-6 (n ≥ 3) **(A)**, anti–IL-6R (n ≥ 6) **(B)**, anti-TNF-α (n ≥ 5) **(C)**, anti-TNF receptor 2 (TNFR2) (n ≥ 5) **(D)**, anti–IL-1α (n ≥ 6) **(E)**, and anti–granulocyte-macrophage colony-stimulating factor (GM-CSF) (n ≥ 5). **(F)** Cellular composition of BALF of mice treated with the following mAbs: anti–IL-6 **(G)**, anti–IL-6 receptor (IL-6R) **(H)**, anti-TNF-α **(I)**, anti-TNFR2 **(J)**, anti–IL-1α **(K)**, and anti-GM-CSF **(L)**. **(M)** Levels of dsDNA in the BALF; *P < 0.05; **P < 0.01, unpaired T test. Poly(I:C) stimulation of IL-6 production in the mouse macrophage cell line RAW264.7 showing the IL-6 concentration in the culture medium **(N)**; **P < 0.01, one-way ANOVA with a *post hoc* Dunnet test. **(O)** Histologic characteristics of mouse lung injury and interstitial pneumonia. Black scale bar = 100 µm; blue scale bar = 50 µm.

We hypothesized that one or more cytokines drive the CSS. To validate this hypothesis, we treated our model mice with neutralizing mAbs against IL-1α, IL-6, and TNF-α, and antibodies that block the IL-6 receptor (IL-6R) and TNF receptor 2 (TNFR2). Neutralizing IL-6 mAbs reduced the production of the IL-6 cytokine in BALF, with no effect on IL-1α and TNF-α (**Figure 4A**). However, IL-6R-blocking mAbs were unable to alter the concentrations of IL-1α, IL-6, and TNF-α in BALF (**Figure 4B**). Anti-TNF-α mAbs reduced the production of the TNF-α cytokine in BALF, with no effect on IL-1α and IL-6 (**Figure 4C**); however, anti-TNFR2 mAbs failed to alter the production of IL-1α, IL-6, or TNF-α (**Figure 4D**). Neutralizing mAbs targeting IL-1α significantly directly reduced the cytokines IL-1α and IL-6, but not TNF-α (**Figure 4E**). Additionally, the concentrations of GM-CSF did not increase in BALF during SARS-CoV-2 challenge, but anti–GM-CSF mAbs did reduce the production of TNF-α (**Figure 4F**). Infiltration of neutrophils causes neutrophilic alveolitis during SARS-CoV-2 challenge. The percentage of neutrophils in BALF were tested after treatment with anti–IL-6 (**Figure 4G**), anti-IL6R (**Figure 4H**), anti–TNF-α (**Figure 4I**), anti-TNFR2 (**Figure 4J**), anti–IL-1α (**Figure 4K**), and anti–GM-CSF (**Figure 4L**) antibodies. The results indicated that the neutralization of cytokines IL-6, TNF-α, and GM-CSF, or the blockage of IL-6R, could reduce the infiltration of neutrophils. Concentrations of dsDNA were not reduced following antibody treatment (**Figure 4M**). Next, we evaluated the production of IL-6 in the culture medium of RAW264.7 cells treated with poly(I:C). Although there was a trend of reduced IL-6 under treatment with 20 µg of each antibody tested, the results showed statistically significant only in 40-µg dose group (**Figure 4N**). Our histologic analysis indicated that treatment with some of the neutralizing or blocking mAbs against inflammatory cytokines partially reduced the neutrophilic inflammation and interstitial edema in the lung; anti-TNFR2 mAbs had no such effect (**Figure 4O**). Therefore, the potential for potent therapeutic efficacy of mAbs applied synergistically can be considered in the clinical setting.

### Evaluation of Targeted Therapy in SARS-CoV-2 Mimic–Induced ARDS

**Figure 5:**
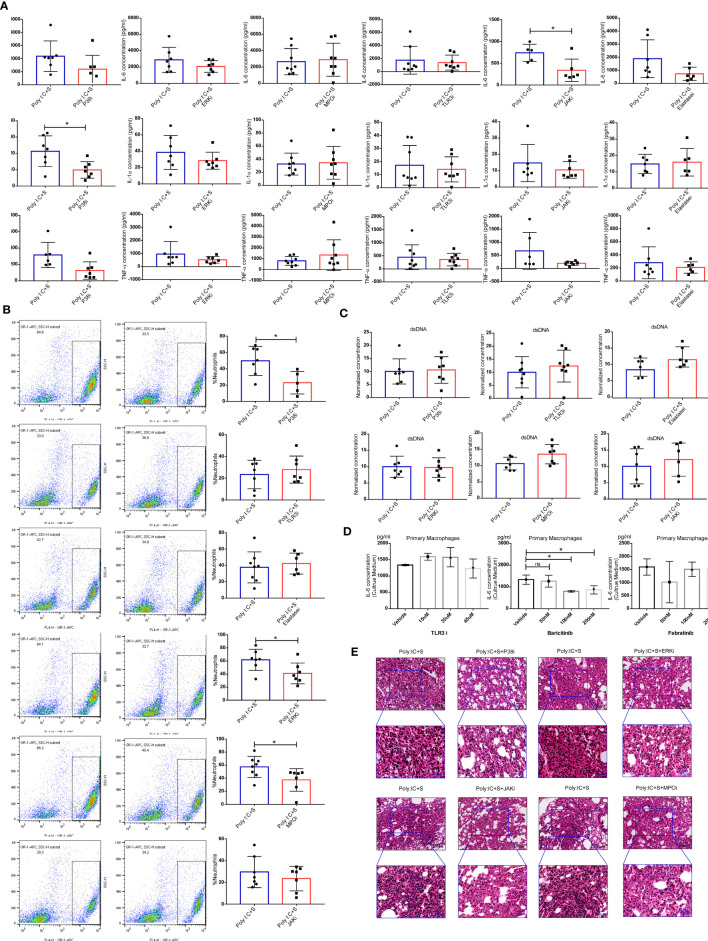
Blocking of inflammation-related signaling pathways. Effect of treatment with the indicated inhibitors on **(A)** the concentrations of IL-6, IL-1α, and tumor necrosis factor (TNF)α in mouse bronchoalveolar lavage fluid (BALF) (each n ≥ 5); **(B)** Cellular composition in BALF; **(C)** levels of dsDNA in BALF; *P < 0.05, unpaired T test. **(D)** poly(I:C) stimulation of IL-6 production in the cell culture medium of mouse primary macrophages; *P < 0.05, ns (non-significant), p > 0.05, one-way ANOVA with a *post hoc* Dunnet test, and **(E)** histologic characteristics of lung injury and interstitial pneumonia. Black scale bar = 100 µm; blue scale bar = 50 µm.

NETs can also be generated by macrophages (18) and strongly contribute to acute lung injury during virus infection (19–21). We hypothesized that the SARS-CoV-2 mimic would stimulate macrophages to produce inflammatory cytokines including IL-1α, IL-6, and TNF-α, and recruit and activate neutrophils. P38 and extracellular signal-regulated kinase (ERK) are the signaling mediators of NETs formation, while myeloperoxidase (MPO) and elastase, as the peroxidase enzymes of neutrophils, are essential for NETs formation (22, 23). The TLR3/dsRNA complex inhibitor can block the interactions between TLR3 and poly(I:C), and the JAK inhibitor blocks the downstream signaling of IL-6. In our study, we found that treatment of mice with inhibitors of P38 and JAK significantly reduced the BALF levels of IL-1α or IL-6, respectively (**Figure 5A**). We also noticed a decrease in the infiltration of neutrophils after treatment with P38, ERK and MPO inhibitors, all of which target the activities of neutrophils (**Figure 5B**). No significant difference was found in the concentrations of dsDNA after treatment with those inhibitors (**Figure 5C**). Furthermore, we performed a cell assay to validate the effect of the TLR3/dsRNA complex and JAK inhibitors on mouse primary macrophages. Treatment with the TLR3/dsRNA complex inhibitor at 45 μM did not reduce IL-6 production (**Figure 5D**). The JAK1/2 inhibitor baricitinib significantly reduced the production of IL-6, but the JAK2 inhibitor febratinib did not (**Figure 5E**). Histologic analysis indicated that treatment with inhibitors of P38, ERK, MPO, and JAK (baricitinib) led to obvious decreases in neutrophilic inflammation and interstitial edema in the lung (**Figure 5F**).

**Figure 6:**
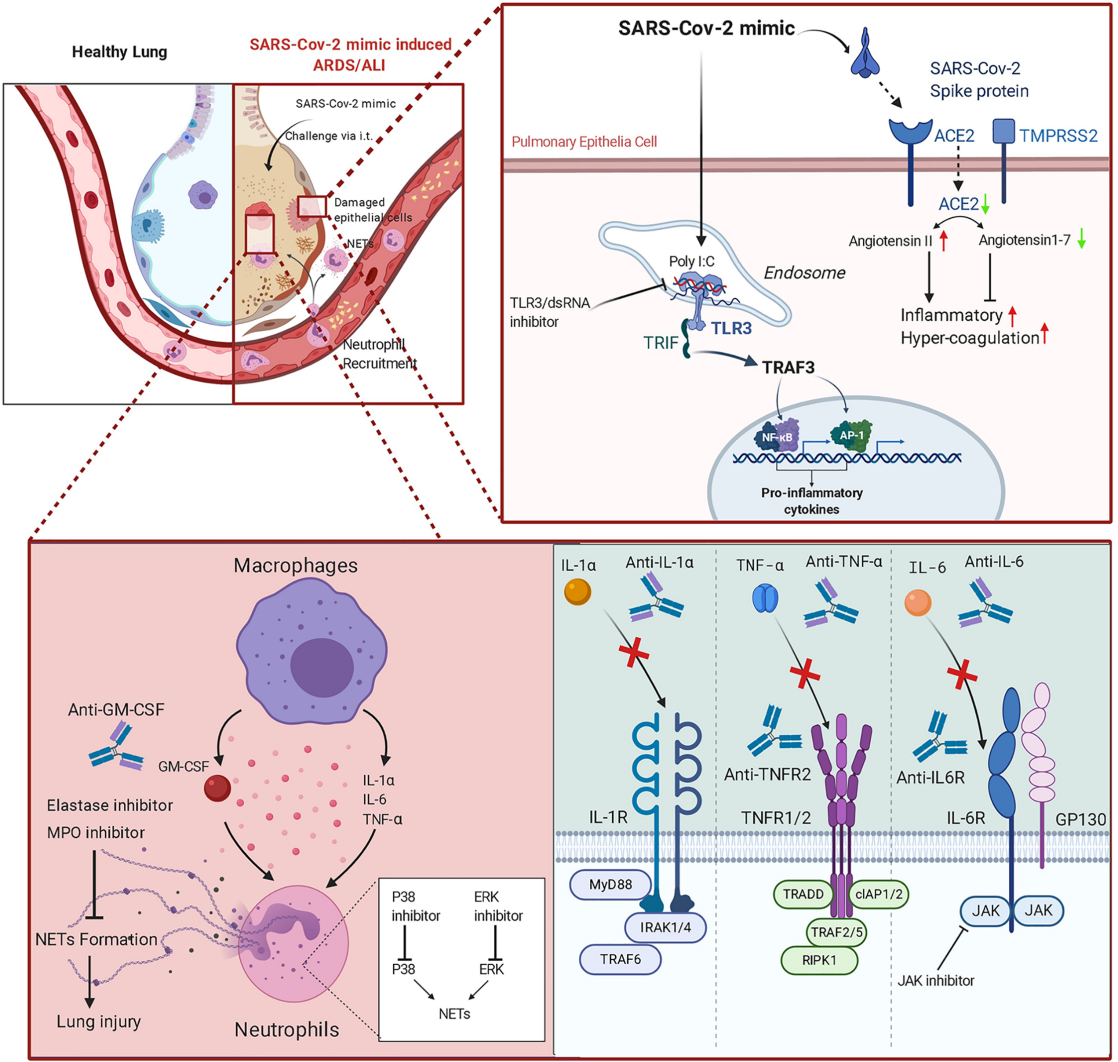
Graphical abstract of the SARS-CoV-2 mimic and potential therapeutic applications of monoclonal antibodies (mAbs) and inhibitors.

A graphical summary of the experimental design of this study is shown in **Figure 6**.

## Discussion

SARS-CoV-2 is a serious threat to global public health. Antiviral drugs including hydroxychloroquine, chloroquine, and remdesivir have not achieved ideal therapeutic effects (24, 25). To investigate the molecular mechanism of SARS-CoV-2–induced ARDS/CSS and find effective strategies to prevent and treat this highly infectious disease, we have developed a murine ARDS model that mimics the pathologic changes observed in COVID-19 patients. SARS-CoV-2 uses ACE2 as the host cell-surface receptor and the serine protease TMPRSS2 to infect humans (1). The receptor-binding domain (RBD) of the viral SP is critical for binding to human ACE2. SARS-CoV-2 shows high affinity for human ACE2, whereas the histidine at position 353 of mouse ACE2 (in place of the lysine in human ACE2) renders it less suitable for the virus-receptor interaction (2). Our SPR sensorgrams revealed that the recombinant spike-ECD protein (rSP) also showed higher affinity for human ACE2 compared with mouse ACE2. Therefore, we increased the dose of SARS-CoV-2 SP used to construct the mouse model. These model mice showed obvious pathologic changes including neutrophilic inflammation and interstitial edema and also displayed significant changes the levels of IL-6 and TNF-α, which are involved in the process of CSS. Our data indicated that this model not only simulates the pathologic changes of COVID-19 but also overcomes the problems of unobvious symptoms and the biological risk of SARS-Cov-2 infection of previous animal models.

Impaired inflammatory responses in patients with severe COVID-19 provide a distinct pattern of COVID-19 progression in the host immune response to SARS-CoV-2 infection; namely, exhausted lymphocytes, a high neutrophil-to-lymphocyte ratio, and CSS (8, 26–29). CSS was found to be the major cause of morbidity in patients with SARS-CoV and MERS-CoV (30), with the presence of IL-6 in the plasma being a hallmark of severe infections. Clinical trials using IL-6 and IL-6R antagonists are underway (31). In our research, we first tested the effects of mAbs against IL-6 and IL-6R in this model; while IL-6 was affected, there were no changes in IL-1α or TNF-α. Importantly, treatment with IL-6 or IL-6R mAbs reduced neutrophil infiltration. This result may indicate that blocking IL-6 signaling offered partial protection from the SARS-CoV-2 mimic–induced neutrophilic alveolitis but did not appear to effectively mitigate CSS in the lung. Furthermore, blocking of JAK-STAT3 signaling might also inhibit IL-6–mediated signal transduction. To block TNF-α signaling, we treated mice with mAbs against TNF-α and TNFR2. TNF exerts its effects *via* stimulation of two different receptors: TNFR1 and TNFR2. TNFR1 is expressed ubiquitously, but TNFR2 is only expressed in certain cell types, including myeloid cells, glial cells and subsets of T cells and B cells. While neutralization of TNF-α *via* mAbs reduced the production of TNF-α in our mouse model, IL-6 and IL-1α were not affected. However, only treatment with mAbs against TNF-α, but not TNFR2, reduced neutrophil infiltration. These results indicated that TNF-α signaling partially reduced neutrophilic alveolitis and protected mice from SARS-CoV-2 mimic–induced CSS. GM-CSF is a cytokine found in high levels in patients with COVID-19, and anti–GM-CSF mAbs were used for the treatment of COVID-19 in a clinical trial (NCT04400929) (32). In our study, mAbs against GM-CSF reduced neutrophil infiltration and decreased TNF-α during the SARS-Cov-2 mimic challenge. Although we did not find GM-CSF enriched in BALF, the anti–GM-CSF treatment showed a protective effect on neutrophilic alveolitis and NETs-induced lung injury.

It was reported that macrophages in the lungs may contribute to inflammation by producing multiple cytokines/chemokines and recruiting inflammatory monocytic cells and neutrophils (26, 33). We tested various signal pathway inhibitors for blocking dysregulated macrophages and neutrophils. The TLR3/dsRNA complex inhibitor blocked the interaction between dsRNA and TLR3 in mouse primary macrophages, but TNF-α in BALF could not be induced by challenge with poly(I:C) alone. During infection with SARS-Cov-2, which has a single strand RNA genome, both TLR3/TLR7 senses the presence of viral RNA (34), the mimic induced CSS in lung may not affect by blocking of TLR3 signaling.

To prevent the sustained activation of neutrophils, we treated the SARS-CoV-2 mimic model mice with P38 inhibitor SB203580, ERK inhibitor tauroursodeoxycholate, MPO inhibitor 4-aminobenzoic hydrazide, and inhibitor of elastase. MPO is used as a marker for the function and activation of neutrophils. Elastase is produced by neutrophils and can mediate lung injury. Inhibitors of MPO and elastase can directly reduce acute lung injury at inflammatory sites (23, 35, 36). The MAPK-p38 signaling pathway plays an essential role in the production of pro-inflammatory cytokines, inhibition of p38 shows promising in COVID-19 therapy (37). Inhibitors of ERK/MAPK signaling decreased the replication of MERS-CoV, and potent as kinase inhibitors to treat COVID-19 (38). MPO and elastase mediated NETs contribute to the severity in COVID-19 and result in ARDS in severe COVID-19 patients (39). Anti-elastase and MPO inhibitors may be available repurposing to prevent ARDS and acute lung injury in COVID-19 (5). In our study, we found that inhibitors of proteins including P38, ERK, MPO, and elastase inhibitors reduced neutrophil infiltration into the mouse lung, but not elastase. We used neutralizing/blocking antibodies and the essential pathway inhibitors of the innate and adaptive immune responses to target the most abundant cytokines, NETs, and other pro-inflammatory factors to prevent SARS-CoV-2–induced CSS and ARDS. Our data indicate the potential for combinations of these neutralizing/blocking antibodies and inhibitors in the treatment of CSS and ARDS. These combinations should be investigated further.

Increased pro-inflammatory cytokines and neutrophils infiltration were present in severe COVID-19 patients, in our murine ARDS model, we noticed the increased pro-inflammatory cytokines IL-6, TNF-α, and IL-1α in BALF. In clinical, a more cytokines network reveals the complicated inflammatory pathogenesis in COVID-19. Some important clinical outcomes, such as IFN-γ and IL-10, indicated that the lack of adaptive immune response to the mimic. Moreover, neutrophils infiltration and NETs formation which paired with clinical outcomes were observed in our model, targeted therapy based on this signaling can be performed in our ARDS model.

In conclusion, we used poly(I:C) + SP to establish a robust, non-infectious, highly safe, time-saving murine model that mimics the pathologic changes of SARS-CoV-2–induced CSS and ARDS, and we identified that TNF-α and IL-6 in BALF, were induced by poly (I:C) and recombinant SP of SARS-CoV-2 in both virus-assisted human ACE2 expression or wild type BALB/c mice. This model can be made available for research to explore the immunopathologic alterations of CSS and ARDS in COVID-19 and new therapies for this deadly disease. The early infected COVID-19 patients may take days to confirm, but only patients with severe COVID-19 might have a CSS. Therefore, it is applicable to start that these interventions for high-risk individuals, such as healthcare workers and household members of an infected individual who should benefit from our research, prevent the COVID-19 to severe.

### Limitations of This Study

About 0.001 EU/μg of endotoxin were detected in the batch of the SP and FC recombinant protein as we used in the animal experiment; in literature, intratracheal instillation of about 100 μg per mice (1 μg LPS = 10^4^EU) of LPS could induce acute lung injury and production of inflammatory cytokines in mouse lung (40, 41). Due to very small dose of endotoxin contained in our solutions of the respective inflammogens, we do not believe that the endotoxin level found in SP and FC is sufficient to cause the observed inflammatory responses in the lung. In our study, we noticed the production of inflammatory cytokines between Saline and SP or FC group shows no significant difference in the animal model.

Nonetheless, as small amounts of endotoxin (e.g., LPS or PepG) can activating macrophages *in vitro* and synergize with other inflammogens (42, 43), we have planed the further animal experiments in LPS-resistant mice strains (e.g., Lps^d^ mouse strains), to exclude that LPS-contamination played a role as the driver of the cytokine storm triggered by the inflammogens used in this study.

## Data Availability Statement

The raw data supporting the conclusions of this article will be made available by the authors, without undue reservation.

## Ethics Statement

The animal study was reviewed and approved by Ethics Review Commission of Zhengzhou University.

## Author Contributions

ZD, TG, and SZ designed the study. TG, SZ, GJ, MS, YFZ, RZ, FM, YQZ, and KW performed the main experiment. HL, MX, and WH collected and analyzed the raw data. ZD, TG and SZ wrote the manuscript. XL, CD, and KL revised the manuscript. All authors contributed to the article and approved the submitted version.

## Funding

ZD acknowledges funding from Zhengzhou collaborative Innovation Major Project, N.O 20XTZX02016.

## Conflict of Interest

The authors declare that the research was conducted in the absence of any commercial or financial relationships that could be construed as a potential conflict of interest.

## Supplementary Material

Supplementary Figure 1 Markers of the endothelial damage. **(A)** the concentrations of soluble mouse E-selectin, VCAM-1 and VEGF in mouse BALF and serum (each n ≥ 4). **P* < 0.05; ***P* < 0.01, ****P* < 0.001, one-way ANOVA with a *post hoc* Bonferroni test.

Supplementary Figure 2 Test of endotoxin contamination. **(A)** The endotoxin contamination in Saline, poly(I:C), FC, and SP in the animal experiment was determined using the Limulus amoebocyte lysate (LAL) assay.
