# Supplementary figures and images for: Cytokine Signature Induced by SARS-CoV-2 Spike Protein in a Mouse Model
Source: Front Immunol. 2021 Jan 28;11:621441. doi: 10.3389/fimmu.2020.621441 (PMC7876321; doi:10.3389/fimmu.2020.621441)

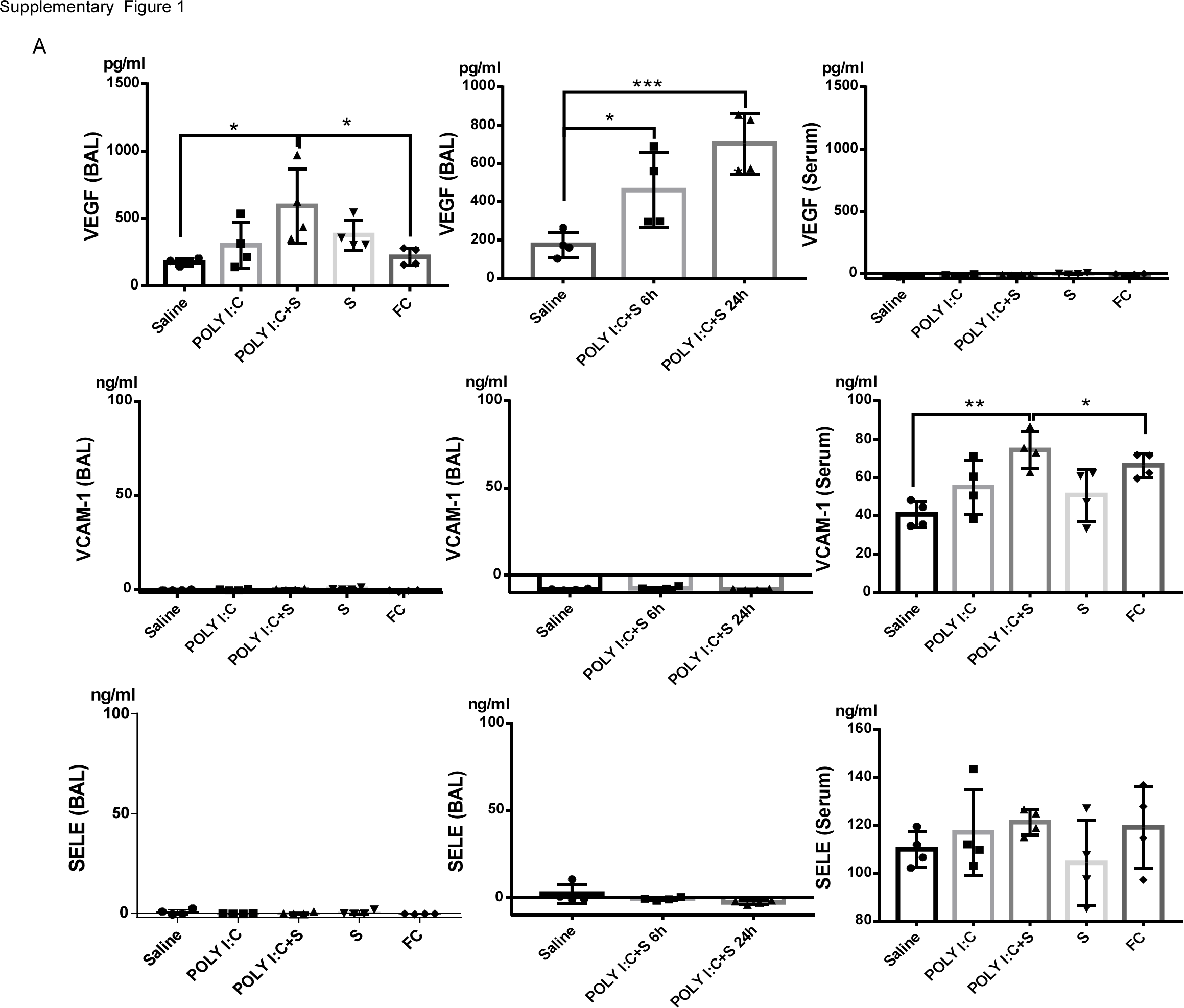

Supplement: Supplementary Figure 1 — Markers of the endothelial damage. (A) the concentrations of soluble mouse E-selectin, VCAM-1 and VEGF in mouse BALF and serum (each n ≥ 4). *P < 0.05; **P < 0.01, ***P < 0.001, one-way ANOVA with a post hoc Bonferroni test. [file Image_1.tif]

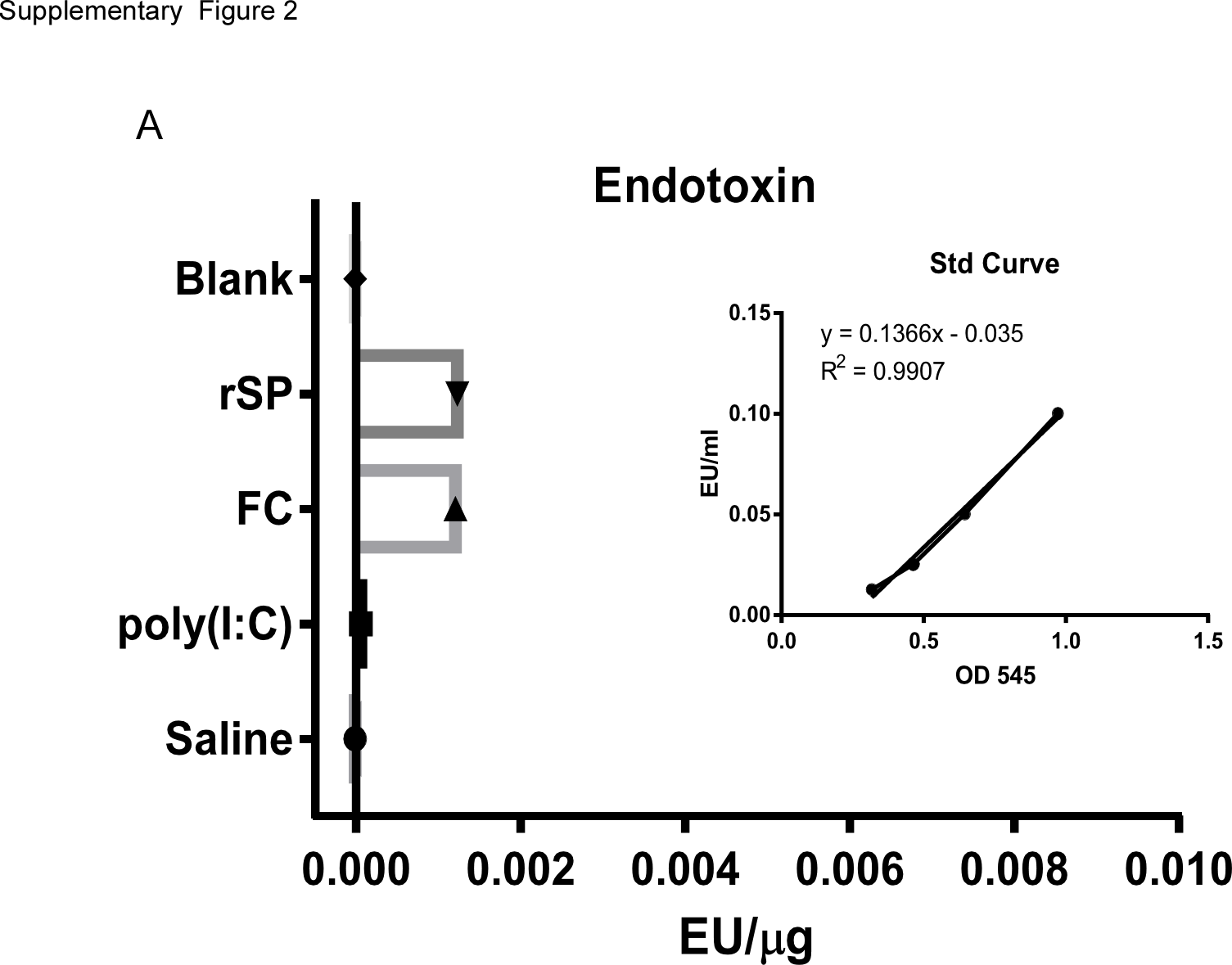

Supplement: Supplementary Figure 2 — Test of endotoxin contamination. (A) The endotoxin contamination in Saline, poly(I:C), FC, and SP in the animal experiment was determined using the Limulus amoebocyte lysate (LAL) assay. [file Image_2.tif]
